# New types of drug use and risks of drug use among men who have sex with men: a cross-sectional study in Hangzhou, China

**DOI:** 10.1186/s12879-018-3091-z

**Published:** 2018-04-17

**Authors:** Lin He, Xiaohong Pan, Ning Wang, Jiezhe Yang, Jun Jiang, Yan Luo, Xingliang Zhang, Xiting Li

**Affiliations:** 1 Zhejiang Provincial Center for Disease Control and Prevention, No. 3399 Bin Sheng Road, Binjiang District, Hangzhou, Zhejiang People’s Republic of China; 2 National Center for AIDS/STD Control and Prevention, Chinese Center for Disease Control and Prevention, Beijing, China; 3 Hangzhou Center for Disease Control and Prevention, Hangzhou, Zhejiang China

**Keywords:** Men who have sex with men, New types of drugs, Rush poppers, HIV, Alcohol

## Abstract

**Background:**

The use of new types of drugs has become more common among men who have sex with men (MSM). The aim of this study was to describe the patterns of the use of new types of drugs, such as methamphetamine, ketamine, ecstasy, and rush poppers, and to examine the factors associated with drug use and HIV infection among MSM in Hangzhou, China.

**Methods:**

This cross-sectional study was conducted between August 2015 and April 2016. We used snowball sampling to recruit MSM; participants were recruited from voluntary counseling and testing centers, baths, bars, Blued (an app for the gay community), QQ groups, clubs, and other types of venues. MSM were included if their previous HIV test results were negative or unknown, or they had not been tested for HIV. MSM were excluded if they were known to be HIV positive before the survey. Face-to-face questionnaires were conducted and a venous blood specimen was drawn from each participant following the interview.

**Results:**

In total, 555 MSM were included; 18.2% (101/555) of the participants had used new types of drugs in the past 3 months. Among the users, 65.3% used single-use rush poppers, while the remainder used ketamine, methamphetamine, ecstasy, or other mixed combinations of drugs. The HIV positivity rate was 14.8% (82/555). Factors associated with increased odds of using new types of drugs in the past 3 months were higher education levels (adjusted odds ratio [AOR] 4.45, 95% confidence interval [CI] 2.12–9.37), having multiple sexual partners (AOR 1.76, 95 CI 1.02–3.05), alcohol use before sexual intercourse (AOR 33.44, 95% CI 10.80–103.50), and seeing friends using new types of drugs.

**Conclusion:**

We revealed the widespread use of new types of drugs, as well as a high diagnosis rate of new HIV infection, among MSM in Hangzhou. The use of new types of drugs was associated with an increased number of sexual partners among MSM; the high-risk sexual behaviors increased the risk of HIV infection. Attention should be given to the use of new types of drugs in MSM, and supervision programs should be strengthened to combat drug use.

## Background

New types of drugs (also known as club or recreational drugs) are psychoactive substances that are chemically synthesized. They can make people excited or inhibited after use, and their long-term continuous use can make people dependent [1]. In 2014, 14 million people were reported to use drugs in China, accounting for approximately 1% of the total population. The use of new types of drugs, namely methamphetamine and ketamine, is growing rapidly. In China, the proportions of the use of new types of drugs among drugs users increased from 28% in 2010 to 49.4% in 2014, exceeding the traditional prevalence of heroin use [2].

The use of new types of drugs among men who have sex with men (MSM) has been a worldwide public health concern [3, 4]. In addition, several studies [5, 6] have shown that the use of new types of drugs could lead to an increased sexual desire, prolonged sexual activity, an increased number of sexual partners and group sex, and the less frequent use of condoms. Previous studies [5, 7] have shown that despite drug use, MSM commonly have multiple sexual partners, anal intercourse, and a lower frequency of condom use. High-risk sexual behaviors caused by new drugs, coupled with the unsafe, high-risk behaviors inherent in MSM populations, increase the risk of HIV infection [8]. Rush poppers are smooth muscle relaxants, which were first used in the United States MSM population to relax the anal sphincter, reduce anal pain, and help MSM achieve rapid sexual arousal in a short period of time. Methamphetamine and rush poppers have been shown to be independent risk factors for HIV infection [9, 10]. A study conducted in Los Angeles [9] reported that, among 6435 MSM participants, a new diagnosis of HIV infection was associated with methamphetamine use compared with nondrug users. Similarly, a multicenter AIDS cohort study revealed that the use of methamphetamine and rush poppers increased the risk of HIV seroconversion [11]. Moreover, the use of certain new types of drugs such as methamphetamine, ecstasy, and poppers might be better predictors of high-risk sexual behavior and HIV seroconversion among MSM [12, 13].

In China, sentinel surveillance of MSM showed that the HIV prevalence increased from 5.7% to 7.8% from 2010 to 2016 [14]; MSM has been the fastest growing population. The use of traditional injection drugs (such as heroin) has historically been very low; however, in recent years, the use of new types of drugs, such as methamphetamine, ecstasy, and rush poppers, has become more common. Previous studies in China have shown that the use of new types of drugs among MSM mainly involved rush poppers [7, 15–17]. The use of rush poppers has been shown to be associated with HIV infection due to risky behaviors, such as multiple sexual partners, commercial sex, group sex, and other high-risk sexual behaviors; 28% of new HIV infections in MSM could be attributed to the use of rush poppers [18]. Studies by Shenyang [19] and Shenzhen [17] showed that the use of new types of drugs was independently associated with an increased risk of HIV infection. Another study that included six cities in China showed that the use of new types of drugs was a risk factor for HIV infection and that MSM who were drug users were more likely to report multiple sexual partners and unprotected sexual intercourse [20]. Another study published online showed that Chinese MSM have a high rate of recreational drug use, including poppers [21]. Moreover, a study in Beijing showed that the use of rush poppers among MSM in the past 3 months was a risk factor for unprotected receptive anal intercourse and for HIV infection among MSM [22].

Hangzhou is located in one of the more developed regions of China; the number of newly diagnosed HIV infections is rapidly increasing on a yearly basis. Approximately 60% of HIV infections occurred in MSM in 2014. A respondent-driven sampling survey in Hangzhou showed that the HIV prevalence among MSM was approximately 8.5% in 2014 [23]. The HIV prevalence among MSM has been 8.5% since 2008. During this same time period, the national HIV prevalence was 4.9% in 2008–2009 [24]. The HIV epidemic in Hangzhou forecasts a future trend for China. The use of new types of drugs increased the risk of unprotected behaviors and the risk of HIV infection. However, there is a lack of data on the use of new types of drugs in Hangzhou among HIV-infected MSM. Although the use of new types of drugs is increasingly popular among MSM, little is known about the patterns of drug use. Therefore, we conducted a survey among HIV-negative MSM from September 2015 to April 2016 to describe the patterns of the use of new types of drugs and to examine the factors associated with drug use and HIV infection among MSM in Hangzhou, China.

## Methods

### Design

This was a cross-sectional study conducted in Hangzhou City, China, between August 10, 2015 and April 30, 2016.

### Sampling methods

We used snowball sampling methods to recruit MSM; MSM were recruited from voluntary counseling and testing centers, baths, bars, Blued (an app for the gay community), QQ groups, clubs, and other types of venues. The initial participants were asked to recruit partners or peers to participate in the survey. After the participants completed the study questionnaires and underwent HIV testing, they were provided with recruitment coupons to incentivize other participants. Using these incentives, the participants then recruited MSM peers from their social network to participate in the survey. Without knowledge of the use of new types of drugs among MSM in Hangzhou, we referred to studies conducted in other regions of China to determine the sample size. These studies showed that the use of new types of drugs ranged from 5% to 25%. *P*-values ≤0.05 and β = 0.1 were considered statistically significant. Thus, the estimated minimum sample size required for this study was 400. The sample size was calculated using WINPEPI (PEPI- for-windows) version 9.5 software.

### Study participants

We included MSM who met the following criteria: were at least 16 years of age; had had anal sex with men in the last 3 months before the study; had previous HIV test results that were negative or unknown; and were willing to provide informed consent to participate in the study. MSM were excluded from the study if they were HIV-infected before the survey; were impaired and could not clearly understand and answer the questions from the investigation questionnaire because of excessive alcohol consumption, poisoning, or other causes, or because they had a mental illness or mental retardation; did not fully understand the process of informed consent or did not give informed consent; did not agree to accept the questionnaire survey and/or serological survey; and refused to take part in the study for any other reasons.

### Questionnaire and data collection

A face-to-face questionnaire survey was conducted, which included questions about socio-demographic characteristics like age, marital status, education, income, sexual orientation, registered residence and living time in Hangzhou, sexual behavior, sexual partner networks in the past 3 months, history of the new types of drugs used, and history of counseling, alcohol use, HIV testing, seeing friends using new types of drugs, and ever known new types of drugs. After the interview, all the participants were tested for HIV, free of charge. The new types of drugs in the study included methamphetamine, ketamine, ecstasy, and rush poppers. All questionnaires were completed at the Love Working Group of Zhejiang, a gay outreach organization in the Xiacheng District (Hangzhou city).

### HIV testing

A venous blood specimen (5 mL) was drawn from each participant following the interview. The HIV screening test was performed using an enzyme-linked immunosorbent assay (ELISA; Anti-HIV ELISA Kit, Zhuhai Livzon Diagnostics Inc., China), and if the result was positive, the specimen was retested using the same ELISA kit as well as another ELISA kit (Anti-HIV ELISA Kit, Beijing Wantai Biological Pharmacy Enterprise Co. Ltd., China). If the results of one or both ELISA tests were positive, the diagnosis was confirmed using Western blot analysis (HIV Blot 2.2, MP Diagnostics, Singapore).

### Statistical analysis

Data were entered into EpiData version 3.1 (http://www.epidata.dk/) via double entry. After the data were cleaned and verified, statistical analysis was performed using SPSS version 19.0. For descriptive analyses, categorical variables are presented as frequencies and proportions, and continuous variables are presented as medians and interquartile ranges (IQR). Differences in the general demographic characteristics were calculated using Student’s *t*-tests, chi-squared (χ^2^) tests, Fisher’s exact test, and Kruskal-Wallis tests. We determined the mean (standard deviation [SD]) and median (IQR) differences. Factors from univariate analysis with *P-*values < 0.10 and/or those previously shown to be associated with the differences in socio-demographic characteristics were included in the multivariate regression models, which were used to calculate adjusted odds ratios (AOR) and their coinciding 95% confidence intervals (CI). The primary outcomes of interest were the new types of drugs used in the past 3 months, HIV infection, and MSM who were HIV infected and had used new types of drugs in the past 3 months. *P*-values ≤0.05 and β = 0.1 were considered statistically significant.

### Ethical considerations

This study was approved the Institutional Review Board of the National Center for AIDS/STD Control and Prevention, China CDC (IRB approval number: X120331209). Written informed consent was provided by all participants prior to completion of the survey. Participants received 20 RMB (approximately 3.2 US$) for their participation in the survey. Participants with positive HIV test results were informed and counseled by the staff of Xiacheng District CDC and received the necessary referral services. This information and counseling was provided at the same location as the interviews (i.e., Love Working Group of Zhejiang).

## Results

In total, 555 MSM were recruited to conduct the survey between August 10, 2015 and April 30, 2016. The median age of MSM was 31 years (IQR 26–-40); 163 (29.4%) MSM were 25–29 years of age and 167 (30.1%) were 30–39 years of age. Moreover, 286 (51.5%) MSM were single and 240 (43.2%) were married or cohabiting, 539 (97.1%) were of Han nationality, 260 (46.8%) were college students, and 235 (42.3%) had an income above 5000 yuan (RMB). The majority of MSM were bisexual (*n* = 329, 59.3%) or homosexual (*n* = 216, 38.9%), were registered residents of another province (*n* = 286, 51.5%), and were living in Hangzhou for over 24 months (*n* = 407, 73.3%, Table 1).

**Table 1:** Socio-demographic characteristics of men who have sex with men who had used new types drugs in past 3 months in Hangzhou, China (*N* = 555)

Variables	N (%)	New types of drug used		χ2	*P*
		No	Yes		
Age y IQR 31(26–40)				19.936	< 0.001
16–24	84(15.1)	65(14.3)	19(18.8)		
25–29	163(29.4)	126(27.8)	37(36.6)		
30–39	167(30.1)	130(28.6)	37(36.6)		
40-	141(25.4)	133(29.3)	8(7.9)		
Marital status				14.032	0.001
Single	286(51.5)	218(48.0)	68(67.3)		
Married/cohabitation	240(43.2)	208(45.8)	32(31.7)		
Divorced/separated	29(5.2)	28(6.2)	1(1.0)		
Ethnicity				0.359	0.549
Han	539(97.1)	440(96.9)	99(98.0)		
Minority	16(2.9)	14(3.1)	2(2.0)		
Education				14.569	0.001
Junior high school and below	149(26.8)	136(30.0)	13(12.9)		
High school and junior college	146(26.3)	120(26.4)	26(25.7)		
College	260(46.8)	198(41.1)	62(61.7)		
Income RMB yuan				8.210	0.016
< 3000	92(16.6)	76(16.7)	16(15.8)		
3000–4999	228(41.1)	198(43.6)	30(29.7)		
5000-	235(42.3)	180(39.6)	83(54.5)		
Sexual orientation				0.467	0.792
Homosexual	216(38.9)	176(38.8)	40(39.6)		
Bisexual	329(59.3)	269(59.3)	60(59.4)		
Heterosexual/uncertain	10(1.8)	9(2.0)	1(1.0)		
Registered residence				0.456	0.796
Local	128(23.1)	103(22.7)	25(24.8)		
Other cities in Zhejiang	141(25.4)	114(25.1)	27(26.7)		
Other provinces	286(51.5)	237(52.2)	49(48.5)		
Living time in Hangzhou (M)				1.726	0.422
< 3	88(15.9)	76(16.7)	12(11.9)		
3–24	60(10.8)	50(11.0)	10(9.9)		
24-	407(73.3)	328(72.2)	79(78.2)		

Of the 555 recruited MSM, 18.2% (101/555) had used new types of drugs in the past 3 months. Among the MSM who used new types of drugs, 15 (2.7%), 13 (2.3%), 9 (1.6%), and 73 (13.2%) used methamphetamine, ketamine, ecstasy, and rush poppers, respectively. Among the 101 MSM who had used new types of drugs, 65.3% (66/101) used rush poppers only, 10.9% (11/101) used ketamine only, 8.9% (9/101) used methamphetamine only, 5.9% (6/101) used ecstasy only, 6.9% (7/101) used rush poppers and methamphetamine, ecstasy, or ketamine, and 2.0% (2/101) used methamphetamine and ecstasy or ketamine. The median year in which MSM first used new types of drugs was 2014 (IQR 2013–2015).

There were significant differences in the age (*P* < 0.001), marital status (*P* = 0.001), educational levels (*P* = 0.001), and income (*P* = 0.016) between MSM who had used new types of drugs in past 3 months and those who had not. There were no significant differences in the other demographic characteristics investigated (*P* > 0.05).

Eighty-two (14.8%) HIV-infected MSM were diagnosed following completion of the survey. The HIV infection rate was 14.5% (66/454) among MSM who had never used new types of drugs in the past 3 months, versus 15.8% (16/101) among MSM who had used new types of drugs in the past 3 months (*P* = 0.012, χ2 = 0.738).

In the past 3 months, among MSM recruited in this study, 4.9% used alcohol before partaking in sexual behaviors, 46.4% had regular homosexual partners, 67.4% had more than 2 sexual partners, 4.3% partook in group sex, 43.1% had anal intercourse for at least 20 min, and 70.3% partook in high-risk sexual behaviors. Overall, 69.2% of MSM had previously been tested for HIV and 13.0% had seen friends using new types of drugs in the past (Table 2).

**Table 2:** Behavioral characteristics of men who have sex with men who had used new types of drugs in past 3 months in Hangzhou, China (N = 555)

Variables	N (%)	New types of drugs used used		χ2	*P*
		No	Yes		
*In past 3 months*					
Alcohol use before sexual				85.549	< 0.001
No	528(95.1)	450(99.1)	78(77.2)		
Yes	27(4.9)	4(0.9)	23(22.8)		
Regular homosexual partner				0.053	0.817
Yes	258(46.5)	210(46.3)	48(47.5)		
No	297(53.5)	244(53.7)	53(52.5)		
Number of sexual behaviors per week				1.572	0.456
< 1	332(59.8)	277(61.0)	55(54.5)		
1	161(29.0)	127(28.0)	34(33.7)		
≥2	62(11.2)	50(11.0)	12(11.9)		
Sexual partners				2.652	0.103
1	181(32.6)	155(34.1)	26(25.7)		
≥2	374(67.4)	299(65.9)	75(74.3)		
Group sex				1.003	0.317
No	532(95.9)	437(96.3)	95(94.1)		
Yes	23(4.1)	17(3.7)	6(5.9)		
Anal intercourse time (mints)				6.536	0.011
< 20	316(56.9)	270(59.5)	46(45.5)		
20-	239(43.1)	184(40.5)	55(54.5)		
High-risk sexual behavior*				0.055	0.815
No	165(29.7)	134(29.5)	31(30.7)		
Yes	390(70.3)	320(70.5)	70(69.3)		
*In past years*					
Ever had HIV testing				0.963	0.326
Yes	384(69.2)	310(68.3)	74(73.3)		
No	171(30.8)	144(31.7)	27(26.7)		
Seeing friends using new types of drug				15.175	< 0.001
Yes	72(13.0)	47(10.4)	25(24.8)		
No	483(87.0)	407(89.6)	76(75.2)		
Ever known new types of drug				0.013	0.911
Yes	360(64.9)	294(64.8)	66(65.3)		
No	195(35.1)	160(35.2)	35(34.7)		

*High-risk sexual behavior meant that MSM have sexual behavior without condom use, MSM had sexual behavior with condom use or had not sexual defined as no high-risk behavior

Compared with MSM who had not used new types of drugs in the past 3 months, MSM who had used new types of drugs in the past 3 months were more likely to have had anal intercourse for at least 20 min (*P* = 0.001), have been tested for HIV (*P* = 0.006), and have seen friends using new types of drugs (*P* < 0.001).

Univariate analysis showed that higher educational levels (odds ratio [OR] 3.28, 95% CI 1.73–6.19), alcohol consumption before sexual activity in the past 3 months (OR 33.17, 95% CI 11.17–98.53), and having seen friends using new types of drugs in the past (OR 4.50, 95% CI 2.62–7.70) were associated with the use of new types of drugs in the past 3 months (Table 3).

**Table 3:** Factors associated with the use of new types of drugs in the past 3 months among men who have sex with men in Hangzhou, China (N = 555)

Variables	Number of new types of drug %(n/N)	Univariate OR(95%CI)	Multivariate AOR(95%CI)
Education			
Junior high school and below	8.7(13/149)	1.00	1.00
High school and junior college	17.8(26/146)	2.27(1.12–4.61)	3.31(1.47–7.45)
College	23.8(62/260)	3.28(1.73–6.19)	4.45(2.12–9.37)
Sexual partners in past 3 months			
1	14.4(26/181)	1.00	1.00
≥2	20.1(75/374)	1.50(0.92–2.43)	1.76(1.02–3.05)
Alcohol use before sexual in the past 3 months			
No	14.8(78/528)	1.00	1.00
Yes	85.2(23/27)	33.17(11.17–98.53)	33.44(10.80–103.50)
Seeing friends using new types of drug			
Yes	34.7(25/72)	4.50(2.62–7.70)	2.58(1.40–4.78)
No	15.7(76/483)	1.00	1.00

Multivariate logistic regression analysis showed that higher educational levels (AOR 4.45, 95% CI 2.12–9.37), more than 2 sexual partners in the past 3 months (AOR 1.76, 95 CI 1.02–3.05), alcohol consumption before sexual activity in the past 3 months (AOR 33.44, 95% CI 10.80–103.50), and having ever seen friends using new types of drugs were factors associated with the use of new types of drugs in the past 3 months (Table 3).

Univariate analysis showed that a registered residence in other provinces (OR 2.94, 95% CI 1.45–5.96), high-risk sexual behaviors in the past 3 months (OR 2.79, 95% CI 1.47–5.30), and never having had an HIV test (OR 4.27, 95% CI 2.63–6.95) were associated with HIV infection in MSM (Table 4).

**Table 4:** Factors associated with HIV infection among men who have sex with men in Hangzhou, China (N = 555)

Variables	HIV infection rate %(n/N)	Univariate OR(95%CI)	Multivariate AOR(95%CI)
Registered residence			
Local	7.8(10/128)	1.00	1.00
Other cities in Zhejiang	10.6(15/141)	1.40(0.61–3.25)	1.39(0.58–3.30)
Other provinces	19.9(57/286)	2.94(1.45–5.96)	2.69(1.30–5.60)
High-risk sexual behavior in the past 3 months*			
No	7.2(12/165)	1.00	1.00
Yes	17.9(70/390)	2.79(1.47–5.30)	2.82(1.45–5.47)
Ever had HIV testing			
Yes	8.6(33/384)	1.00	1.00
No	28.7(49/171)	4.27(2.63–6.95)	4.31(2.62–7.11)

*High-risk sexual behavior meant that MSM have sexual behavior without condom use, MSM had sexual behavior with condom use or had not sexual defined as no high-risk behavior

Multivariate logistic regression analysis also confirmed that a registered residence in other provinces (AOR 2.69, 95% CI 1.30–5.60), high-risk sexual behaviors in the past 3 months (AOR 2.82, 95% CI 1.45–5.47), and never having had an HIV test (AOR 4.31, 95% CI 2.62–7.11) were risk factors for HIV infection in MSM (Table 4).

Univariate and multivariate analysis showed that the use of new types of drugs and the use of rush poppers individually were not associated with HIV infection in MSM.

Univariate analysis showed that having a registered residence in other provinces (OR 8.67, 95% CI 1.06–70.67), having anal intercourse for at least 20 min in the past 3 months (OR 4.44, 95% CI 1.18–16.70), and never having had an HIV test (OR 6.67, 95% CI 2.13–20.91) were associated with HIV infection in MSM (Table 5).

**Table 5:** Factors associated with HIV infection among men who have sex with men who used new types of drugs in the past 3 months in Hangzhou, China (*N* = 101)

Variables	HIV infection rate %(n/N)	Univariate OR(95%CI)	Multivariate AOR(95%CI)
Registered residence			
Local	4.0(1/25)	1.00	1.00
Other cities in Zhejiang	7.4(2/27)	1.92(0.16–22.58)	3.83(0.26–56.80)
Other provinces	26.5(13/49)	8.67(1.06–70.67)	18.77(1.73–203.55)
Anal intercourse time (mints)			
< 20	6.5(3/46)	1.00	1.00
20-	23.6(13/55)	4.44(1.18–16.70)	7.61(1.45–39.99)
Ever had HIV testing			
Yes	9.2(6/74)	1.00	1.00
No	37.0(10/27)	6.67(2.13–20.91)	13.16(2.99–57.91)

Multivariate logistic regression analysis showed that having a residence registered in other provinces (AOR 18.77, 95% CI 1.73–203.55), having anal intercourse for more than 20 min in the past 3 months (AOR 7.61, 95% CI 1.45–39.99), and never having had an HIV test (AOR 13.16, 95% CI 2.99–57.91) were risk factors for HIV infection in MSM who had used new types of drugs in the past 3 months (Table 5).

## Discussion

Our study revealed the widespread use of new types of drugs among MSM and the high frequency of newly diagnosed HIV infections. We showed that the most commonly used new types of drugs among MSM in Hangzhou were rush poppers; some MSM used ketamine and methamphetamine, whereas others mixed a variety of drugs. Compared with the non-users, the users of new types of drugs were more likely to have anal intercourse for at least 20 min, consume alcohol before sexual activity, and have previously seen friends using new types of drugs.

The HIV status of the participants was not known before the survey; following testing, we found that 14.8% of participants were HIV positive. The reason for this might be that our participants, who were included if they had had anal sex in the last 3 months, had high level of sexual activity. In addition, our study confirmed that the use of new types of drugs increased the frequency of unprotected sexual activities among MSM; risky sexual behaviors might increase the risk of HIV infection [20, 25]. We found that the rate of new types of drugs used in the past 3 months was 18.2% and the rate of the use of rush poppers was 13.1%. Compared with previous studies in China, the rate of the use of new types of drugs or rush poppers in Hangzhou was lower than in other cities (Guangzhou, 14.8% [7]; Shenyang, 26.5% [20]; Nanjing, 19.3% [15]; Beijing, 26.8% [26]; and online, 21.3% [21]), and higher than that in Shenzhen (12.7%) [17]. This could be because rush poppers were excluded from the list of controlled drugs in China after 2013; therefore, they could be easily obtained from the internet [19, 21]. The widespread use of new types of drugs in the MSM population and the high HIV infection rate in Hangzhou are a threat to the prevention and control of HIV in the MSM population. Our findings suggest that the mechanisms already in place to control and prevent the use of new types of drugs among MSM should be improved. However, we believe that the new types of drugs should be included in the sentinel surveillance system because of risky MSM behaviors and the potential risk of HIV.

We found that rush poppers accounted for 65.3% of the new drugs used among MSM in the past 3 months; the other drugs commonly used were ketamine (10.9%), methamphetamine (8.9%), ecstasy (5.9%), and other combinations of mixed drugs (8.9%). Our findings are similar to those from a study among American MSM, in which poppers were the most commonly used drugs (61%), followed by methamphetamine (23%) and cocaine (14%). Contrary to a study [19] in Shenyang, 17.2%, 3.4%, and 2.8% of MSM had a history of using methamphetamine, ketamine, and ecstasy in the current study, respectively; this was higher than the findings described by Zhao et al. [21], which showed a use rate of 2.4% for crystal meth and 1.0% for ecstasy among MSM. Thus, the frequency of the use of other drugs in the past 3 months among MSM in Hangzhou was much higher than that among MSM in Shenyang. This might be due to the fact that Hangzhou is located on the coast and is more developed economically; the MSM in Hangzhou may be more able to afford new types of drugs through their social networks. With higher educational levels and better economic opportunities, the MSM in Hangzhou have a greater ability to purchase new types of drugs. Thus, attention should be given to the use of new types of drugs, including ketamine and methamphetamine, in MSM. Programs should provide support to MSM at high risk and provide interventions to mitigate the risk of HIV infection.

The present study showed that alcohol consumption before sexual intercourse in the past 3 months was a risk factor for the use of new types of drugs. After drinking alcohol, the consciousness is blurred and judgment is decreased; previous studies have shown that heavy alcohol consumption was independently associated with serodiscordant unprotected anal sex [27–29]. A study conducted in Beijing showed that alcohol consumed during sex (AOR 1.32 95% CI 1.10–1.60) was associated with the use of poppers, and was independently associated with sexual risk among MSM [30]. The overall rates of alcohol consumption are high among Chinese MSM. Consistent with the findings of our study, a study conducted in Spain reported that a high proportion of MSM used alcohol and drugs before and during sex [31]. Studies in developed and developing countries have found that alcohol consumption or the frequency of drinking increased HIV risk behaviors and the risk of acquiring HIV and other sexually transmitted diseases among MSM [32, 33]. Based on this, we suggest that more attention should be given to MSM who consume alcohol before sexual activities, and there should be more targeted interventions to reduce the frequency of new types of drugs use in MSM.

Furthermore, having previously seen friends using drugs was a risk factor associated with the use of new types of drugs. Consistent with our results, a study in Shanghai showed that approximately 28.6% of adults who used new types of drugs for the first time were influenced by peers [34]. Another study revealed that the main reasons for using drugs were peer influence and seeking to draw attention or cause controversy [35]. Interestingly, a previous study suggested that drug use and sexual behaviors among Chinese MSM differed widely based on the sale of sex and unprotected sex [36]. We suggest that health education should be provided to users of new types of drugs to make them aware of the dangers of using such drugs. This may also influence their social network partners and friends to reduce their use of new types of drugs.

Our study had several limitations. Firstly, this was a cross-sectional study; therefore, the causal factors associated with the use of new types of drugs and HIV infection could not be determined. Moreover, it could not be determined whether alcohol consumption before sexual activity led to drug use, or whether drug use led to alcohol consumption. The study placed all types of new drugs into one category. This may increase the analytical sample size; however, scientifically, it masks important information. Rush poppers require special attention. Secondly, we did not use probabilistic sampling; instead, we used snowball sampling to recruit MSM. Although the MSM were recruited from a variety of settings, we could not determine the rate of the use of new types of drugs and HIV infection for all MSM in Hangzhou. Furthermore, we only recruited MSM whose previous HIV test results were negative or unknown and all of the HIV infection cases identified during the study period were newly diagnosed; however, we could not confirm if HIV infection was the result of the use of new types of drugs. Lastly, the use of new types of drugs is illegal in China; therefore, some participants might not have reported their use of new types of drugs. Therefore, the frequency of use might have been underestimated.

## Conclusion

We noted high proportions of the use of ketamine, methamphetamine, and other drugs and a high rate of newly diagnosed HIV infection among MSM in Hangzhou. The use of new types of drugs was associated with an increased number of sexual partners among MSM; the high-risk sexual behaviors increased the risk of HIV infection. We suggest that attention should be given to the use of new types of drugs in MSM, and supervision programs should be strengthened to combat the use of new types of drugs.

## Abbreviations

χ2: chi squared test
AOR: adjusted odds ratio
CI: confidence interval
IQR: interquartile range
MSM: men who have sex with men
VL: viral load
